# Genomics and Virulence of *Fonsecaea pugnacius*, Agent of Disseminated Chromoblastomycosis

**DOI:** 10.3389/fgene.2020.00822

**Published:** 2020-08-04

**Authors:** Amanda Bombassaro, Gabriela X. Schneider, Flávia F. Costa, Aniele C. R. Leão, Bruna S. Soley, Fernanda Medeiros, Nickolas M. da Silva, Bruna J. F. S. Lima, Raffael J. A. Castro, Anamélia L. Bocca, Valter A. Baura, Eduardo Balsanelli, Vania C. S. Pankievicz, Nyvia M. C. Hrysay, Rosana H. Scola, Leandro F. Moreno, Conceição M. P. S. Azevedo, Emanuel M. Souza, Renata R. Gomes, Sybren de Hoog, Vânia A. Vicente

**Affiliations:** ^1^Microbiology, Parasitology and Pathology Post-graduation Program, Department of Basic Pathology, Federal University of Paraná, Curitiba, Brazil; ^2^Engineering Bioprocess and Biotechnology Post-graduation Program, Department of Bioprocess Engineering and Biotechnology, Federal University of Paraná, Curitiba, Brazil; ^3^Pharmacology Post-graduation Program, Department of Pharmacology, Federal University of Paraná, Curitiba, Brazil; ^4^Graduation in Biology Sciences, Federal University of Paraná, Curitiba, Brazil; ^5^Department of Cell Biology, University of Brasilia, Brasilia, Brazil; ^6^Department of Biochemistry, Federal University of Paraná, Curitiba, Brazil; ^7^Service of Neuromuscular and Demyelinating Diseases, Complex Histochemistry-Immunity Laboratory, Hospital of Clinics, Federal University of Paraná, Curitiba, Brazil; ^8^Department of Medicine, Federal University of Maranhão, São Luís, Brazil; ^9^Center of Expertise in Mycology of Radboud University Medical Center/Canisius Wilhelmina Hospital, Nijmegen, Netherlands

**Keywords:** black fungi, cerebral infection, genome assembly, virulence, dissemination, pathology, chromoblastomycosis, neurotropism

## Abstract

Among agents of chromoblastomycosis, *Fonsecaea pugnacius* presents a unique type of infection because of its secondary neurotropic dissemination from a chronic cutaneous case in an immunocompetent patient. Neurotropism occurs with remarkable frequency in the fungal family Herpotrichiellaceae, possibly associated with the ability of some species to metabolize aromatic hydrocarbons. In an attempt to understand this new disease pattern, were conducted genomic analysis of *Fonsecaea pugnacius* (CBS 139214) performed with *de novo* assembly, gene prediction, annotation and mitochondrial genome assembly, supplemented with animal infection models performed with *Tenebrio molitor* in *Mus musculus* lineages BALB/c and C57BL/6. The genome draft of 34.8 Mb was assembled with a total of 12,217 protein-coding genes. Several proteins, enzymes and metabolic pathways related to extremotolerance and virulence were recognized. The enzyme profiles of black fungi involved in chromoblastomycosis and brain infection were analyzed with the Carbohydrate-Active Enzymes (CAZY) and peptidases database (MEROPS). The capacity of the fungus to survive inside *Tenebrio molitor* animal model was confirmed by histopathological analysis and by presence of melanin and hyphae in host tissue. Although *F. pugnacius* was isolated from brain in a murine model following intraperitoneal infection, cytokine levels were not statistically significant, indicating a profile of an opportunistic agent. A dual ecological ability can be concluded from presence of metabolic pathways for nutrient scavenging and extremotolerance, combined with a capacity to infect human hosts.

## Introduction

Melanized fungi that are known as ‘black yeasts and relatives’ and belonging to the family Herpotrichiellaceae (order Chaetothyriales) are associated with different clinical pictures such as mycetoma, phaeohyphomycosis, and chromoblastomycosis (Cañete-Gibas and Wiederhold, 2018). Chromoblastomycosis starts at the inoculation site of the etiological agent, leads to chronic acanthosis, and, triggered by the host’s immune response, develops fungal structures known as muriform cells (Vicente et al., 2017). Phaeohyphomycosis can be distinguished from other infectious syndromes by tissue invasion with pigmented hyphae (Thomas et al., 2018) and is often associated with necrosis.

Significant differences in pathogenicity and virulence between the main clinical species *Cladophialophora bantiana*, *Exophiala dermatitidis, Fonsecaea pedrosoi*, and *Rhinocladiella mackenziei* on the one hand, and closely related environmental species on the other, have been reported. These species can grow at human body temperature or higher and are able to cause systemic or disseminated disease, while many others, if causing infection, remain subcutaneous (Seyedmousavi et al., 2014; Teixeira et al., 2017). Cerebral infections by black fungi are characterized by abscesses with hyphae in tissue and are therefore classified as phaeohyphomycosis. Such infections often lead to death of the patient despite combined treatment with antifungal drugs and surgery (de Azevedo et al., 2015; Arcobello and Revankar, 2020).

Infection of humans by members of Herpotrichiellaceae is enabled by their stress tolerance and adaptability in their natural, environmental niche, the human host not being a preferential habitat (Gostinčar et al., 2018). The agents are saprobes in mostly still unclarified micro-habitats, and decompose organic matter for nutrition (Vicente et al., 2017). The high adaptability and invasive potential explain the relatively high frequency in animal hosts despite a low environmental occurrence. This potential for infection appears to be polyphyletic within the family Herpotrichiellaceae, as it differs between species (Vicente et al., 2017).

*Fonsecaea* sibling species differ significantly in their ecology and potential of infection (Vicente et al., 2014). Some, such as *Fonsecaea pedrosoi* and *F. nubica*, are recognized etiologic agents of chromoblastomycosis in human hosts, having an ability to form muriform cell in tissue, while *F. monophora* and *F. pugnacius* may also be involved in disseminated infection with hyphal growth in the brain (de Hoog et al., 2004; Tanabe et al., 2004; Najafzadeh et al., 2009, 2011; Vicente et al., 2012; de Azevedo et al., 2015). Nine cases of primary brain infection by these species have been confirmed (Lucasse et al., 1954; Nobrega et al., 2003; Surash et al., 2005; Takei et al., 2007; Koo et al., 2010; Raparia et al., 2010; Doymaz et al., 2015; Varghese et al., 2016; Helbig et al., 2018). *Fonsecaea pugnacius* is exceptional by combining features of chromoblastomycosis and secondary neurotropic dissemination. The single strain known to date of the species presented muriform cells in subcutaneous tissue, but hyphae in the cerebrum (de Azevedo et al., 2015). This duality of local and invasive morphologies has not been observed in any other species associated with chromoblastomycosis or brain infection, suggesting that *Fonsecaea pugnacius* presents a unique pathogenic profile different from that of *Cladophialophora bantiana*, the main agent of human brain infection which presumably follows a pulmonary route (Ozgun et al., 2019).

In the present study, we sequenced the genome of the type strain of *F. pugnacius*, CBS 139214, and performed genomic analysis in order to identify the relation between black fungi and neurotropism. In addition, we compared the enzymatous gene profile of *F*. *pugnacius* to other previously sequenced neurotropic species, including *C. bantiana*, *E. dermatitidis* and *R. mackenziei*. To obtain more insight into virulence, we evaluated animal infection models by *F. pugnacius*, using strain CBS 139214 isolated from a cutaneous lesion of a patient with disseminated neurotropic infection.

## Materials and Methods

### Genomic DNA Extraction, Sequencing and Assembly

*Fonsecaea pugnacius* strain CBS 139214 (type) originating from a skin lesion (de Azevedo et al., 2015) was obtained from the reference collection of Westerdijk Fungal Biodiversity Institute, Utrecht, Netherlands. The strain was grown in Sabouraud liquid medium during 7 days at 28°C for DNA extraction according to Vicente et al. (2008) using cetyltrimethylammonium bromide (CTAB) and phenol-chloroform/isoamyl alcohol and the Microbial DNA UltraClean^TM^ kit for purification. The Nextera kit (IlluminaTM) and Ion Plus Fragment Library Kit (Thermo Fisher Scientific) were used to prepare the DNA libraries for sequencing based on the producer’s guidelines. FastQC^1^ was used for quality control analyses of sequence reads generated. The SPAdes assembler v3.10.0 (Bankevich et al., 2012) and FGAP (Piro et al., 2014) were applied for *de novo* assembly and gap closure, respectively. The genome assembly was evaluated by BUSCO v4.0.2 using the ‘chaetothyriales_odb10’ dataset (Seppey et al., 2019) and the Bowtie2 program was used for assembly coverage measure (Langmead and Salzberg, 2012).

### Gene Prediction, Annotation and Genomic Analysis

GeneMark-ES v4.39 (Besemer et al., 2001) was applied to predict the protein-coding genes using default parameter and RAFTS3 (Vialle et al., 2016) for automatic annotation with best hits comparison with self-score cutoff 0.5 using our internal database of sequences of *Fonsecaea* ssp. and related species from the Chaetothyriales (Vicente et al., 2017; Moreno et al., 2018). *F. pugnacius* functional characteristics were determined with GO enrichment analyses at a significance level of ≤0.05 according to Ashburner et al. (2000) using the InterProScan5 (Quevillon et al., 2005) to access the protein domain families. Phylogenomic trees based on orthologous clusters were obtained and generated using ORTHOFinder (Emms and Kelly, 2018). A phylogenomic tree was inferred for each and all-orthogroup trees were resolved by the OrthoFinder duplication-loss coalescent model (Emms and Kelly, 2018) and the final tree was constructed using the STAG method, present in the OrthoFinder pipeline (Emms and Kelly, 2018). The enzymatous gene profile of *F. pugnacius* and other melanized fungi causing brain infection (*C. bantiana, E. dermatitidis, F. monophora, Verruconis gallopava*, and *R. mackenziei*) were predicted with CAZY (Cantarel et al., 2009) and MEROPS databases (Rawlings et al., 2016). In addition, numbers, classes and similarities were analyzed using an all-vs.-all similarity 40% ≥ search and *e*-value of 10^–4^ (Vicente et al., 2017).

### Mitochondrial Genome Assembly and Annotation

The SPAdes v3.6.2 program (Bankevich et al., 2012) was used for assembly and mappingd the mitochondrial genome from the *F. pugnacius* sequencing reads previously aligned against the complete mtDNA of *Fonsecaea pedrosoi* CBS 271.37. Mitochondrial genome annotations were done based on Vicente et al. (2017) and Moreno et al. (2018) using SILA (Vialle, 2013) and the final figure was produced by the software package Circos (Krzywinski et al., 2009).

### Virulence Assays in Animal Models

#### *Tenebrio molitor* Infection

A *Tenebrio molitor* larval model was used to evaluate the virulence potential based on Fornari et al. (2018) using parameters of survival and melanization after infection. Larvae were inoculated with 1 × 10^6^ cells/mL in PBS solution above the legs and the ventral portion sterile PBS solution and SHAM without physical damage (no treatment) as negative control, using 10 larvae per group of inoculation, in triplicate. The larvae were kept in darkness at 37°C, mortality was monitored daily for 10 days and dead larvae were collected at 4, 24, 72, 168, and 240 h post infection omitting the pupae in the calculation (Scorzoni et al., 2013). Survival curves were plotted and statistical analyses were performed using the Log-rank (Mantel–Cox) test with Graph Pad Prism software and statistical differences were set at *p* < 0.05 according to Maekawa et al. (2015) and Vicente et al. (2017). Melanization was determined measuring the OD at 405 nm (Scorzoni et al., 2013; Perdoni et al., 2014).

Fungal burden and histological analysis of infected caterpillars was performed according to Fornari et al. (2018). The samples were homogenized in PBS solution with a TissueLyser (Qiagen, Hilden, Germany), inoculated on Mycosel agar at 30°C for 14 days and the number of colony forming units (CFUs) of fungal per mL of solution estimated with some colonies re-isolated and sequenced to confirm the species ID. Moreover, the caterpillar samples were embedded in Adracanth gum solution (Fornari et al., 2018), immersed in liquid nitrogen and sectioned by steel blades in a cryostat (Leica CM 1850, Wetzlar, Germany), stained with hematoxylin and eosin (HE) and observed with Axio Imager Z2 (Carl Zeiss, Jena, Germany) equipped with Metafer 4/VSlide automated capture software (Metasystems, Altlussheim, Germany).

#### Murine Infection

Fungal burden and cytokine production evaluation was performed using immunocompetent mice as model, according Bocca et al. (2006), Badali et al. (2011), Rodrigues et al. (2015), and Schneider et al. (2019). The animals selected were male Balb/c (6–8 weeks) and C57/BL6 mice, maintained under standard laboratory conditions with controlled temperature (23–25°C) with water and food *ad libitum*, according to recommendations of the Federal University of Paraná Ethics Committee (current approval certificate 1002).

The experiments were performed in triplicate using groups of six animals infected with *F. pugnacius* CBS 139214 and one negative control inoculated with sterile phosphate-buffered saline (PBS according to described by Fornari et al. (2018) and Schneider et al. (2019). The animals were infected intraperitoneally or intradermally (per hind footpad) with 100 μL of 1 × 10^6^ propagules or sterile PBS and were monitored weekly and sacrificed at 7, 14, and 21 days post-infection using CO_2_ anesthesia in an appropriate chamber (Fornari et al., 2018). Brain, lung, liver, kidney, spleen, footpad, and blood were aseptically collected for analysis.

For fungal burden determination, samples tissues were weighed, homogenized and diluted in PBS for culture as described by Vicente et al. (2017). Results were expressed as number of CFU ± standard error of mean (SEM) per gram of fresh tissue, counting colonies from the seventh day until the 15th day. Cytokine production was measured from homogenized tissue obtained from infected and non-infected (healthy) animals by ELISA (Vicente et al., 2017). The cytokines interleukin-1β (IL-1β), TNF-α, interleukin-6 (IL-6) and monocyte chemoattractant protein-1 (MCP-1/Ccl2) were measured with kits purchased from eBioscience and used according the manufacturer’s instructions. Results were expressed as pg of cytokine ± standard error of mean (SEM) per 100 milligrams of tissue. The infected tissue samples were fixed in 10% formalin, dehydrated in alcohol, and embedded in paraffin (Fornari et al., 2018). Serial 5-μm sections were stained with hematoxylin and eosin to visualize pathogen morphology.

## Results and Discussion

### *De novo* Assembly

The genome sequencing of *F. pugnacius* CBS 139214 was performed using Illumina MiSeq and Ion proton producing 5,424,908 paired-end reads and 1,853,059 mate-paired reads, respectively. The final high-quality assembly comprised 386 contigs with 34,872,293 bp and 52% of G + C content. The genome size estimated is 34.8 Mb with average coverage of 48.75X and using 97% of the reads for draft assembling. The genome completeness, checked using BUSCO, revealed that the assembly had 98.8% completeness. A total of 6188 complete BUSCO genes were found, including 6176 being single-copy BUSCOs, of the 6265 BUSCO groups searched, 12 (0.2%) were duplicated BUSCO genes, 39 (0.6%) were fragmented BUSCOs and 38 (0.6%) represented missing BUSCOs. Sequencing data were submitted to GenBank (accession number WJFF00000000). In addition, 12,217 protein-coding genes and 35 tRNAs were predicted for *F. pugnacius* (Table 1). Expected values for *Fonsecaea* siblings were between 11,681 in *F. nubica* and 12,527 in *F. pedrosoi* (Vicente et al., 2017).

**TABLE 1:** *Fonsecaea pugnacius* genome data assembly and related species of Herpotrichiellaceae.

**Species**	**Strains**	**Genome size (Mbp)**	**GC content (%)**	**Number of proteins**	**GenBank genome access**
*Fonsecaea pugnacius*	CBS 139214	34.8	52	12,217	WJFF00000000
*Fonsecaea pedrosoi*	CBS 271.37	34.69	52.4	12,527	PRJNA233314
*Fonsecaea monophora*	CBS 269.37	35.23	52.2	11,984	LVKK00000000.1
*Fonsecaea nubica*	CBS 269.64	33.79	52.5	11,681	LVCJ00000000.1
*Fonsecaea multimorphosa*	CBS 102226	33.45	52.6	12,369	PRJNA233317
*Fonsecaea multimorphosa*	CBS 980.96	33.39	52.60	11,804	LVCI00000000.1
*Fonsecaea erecta*	CBS 125763	34.75	53.1	12,090	LVYI00000000.1
*Cladophialophora immunda*	CBS 834.96	43.03	52.8	14,033	JYBZ00000000.1
*Cladophialophora bantiana*	CBS 173.52	36.72	51.3	12,762	JYBT00000000.1
*Cladophialophora carrionii*	CBS 160.54	28.99	54.3	10,373	PRJNA185784
*Cladophialophora yegresii*	CBS 114405	27.90	54.0	10,118	AMGW00000000.1
*Capronia epymices*	CBS 606.96	28.89	53.4	10,469	GCA_000585565.1
*Capronia coronata*	CBS617.96	25.81	52.7	9,231	AMWN00000000.1
*Exophiala dermatitidis*	NIH/UT8656	26.38	51.47	9,578	GCA_000230625.1
*Rhinocladiella mackenziei*	CBS 650.93	32.47	50.4	11,382	JYBU00000000.1
*Coniosporium apollinis*	CBS 100218	28.65	52.1	9,308	AJKL00000000.1
*Exophiala mesophila*	CBS 40295	29.27	50.40	10,347	GCA_000836275.1
*Exophiala aquamarina*	CBS 119918	41.57	48.3	13,118	AMGV00000000.1

**FIGURE 1:**
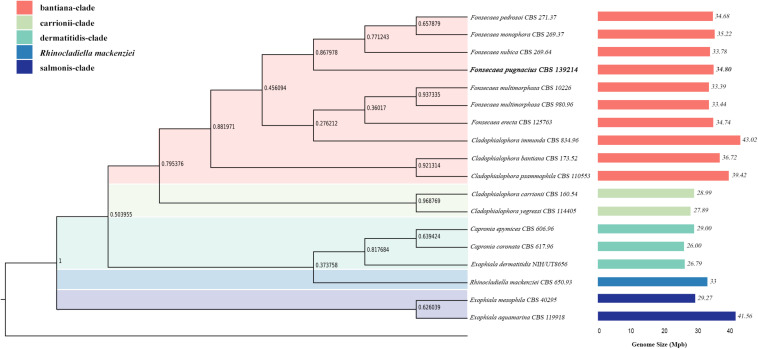
Phylogenomic tree based on the concatenated alignment of genomes of Herpotrichiellaceae family. Species names are given between phylogenomic tree and genome size bars. The genomes size is indicated and the colors are representing the clades of Chaetothyriales summarized in colored boxes.

Comparing *F. pugnacius* genome size of 34.8 Mb with related species of Herpotrichiellaceae (Table 1), some differences were observed. The *Cladophialophora immunda* genome is nearly 8.15 Mb larger, while the *Exophiala dermatitidis* genome is 8.43 Mb smaller, being the smallest genome in the family with 26.37 Mb (Teixeira et al., 2017). Genome sizes within *Fonsecaea* were similar, i.e., between 33.39 and 35.23 Mb (Vicente et al., 2017). Judging from the phylogenomic tree (Figure 1), *F. pugnacius* clustered with the clinical representatives of *Fonsecaea*, in accordance with previous phylogenetic analyses of *Fonsecaea* and *Cladophialophora* based on internal transcribed spacer (ITS), partial beta-tubulin protein-coding gene (BT2) and cell division cycle 42 (CDC42) sequences performed by de Azevedo et al. (2015).

**FIGURE 2:**
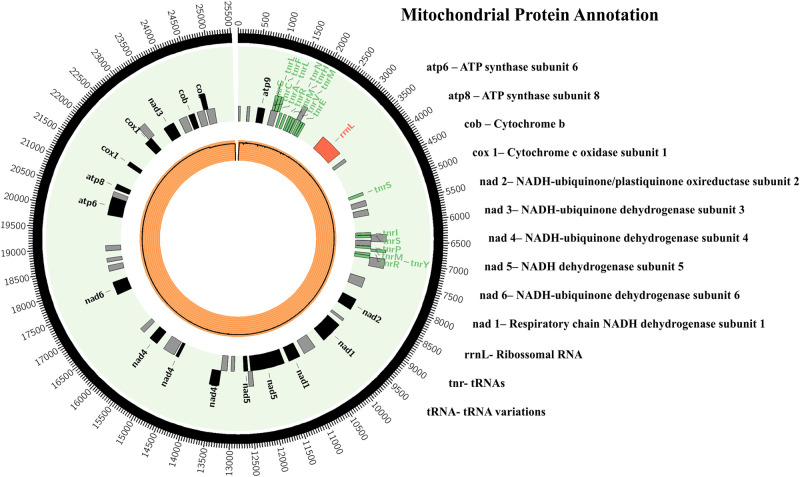
*Fonsecaea pugnacius* CBS 139214 mtDNA. Rectangles represent annotated genes: in red the rRNAs; in green the tRNAs; in black the other genes and orange inner circle shows reads coverage.

The mitochondrial genome was assembled and 3 contigs were obtained with a total of 25,098 bp and a GC% of 25.49; the largest contig was 14,011 bp. There were 44 proteins in the mtDNA, i.e., 26 hypothetical proteins and 18 with known function (Figure 2). The proteins encoded by mitochondrial genomes of herpotrichiellaceous species were very similar, most being proteins involved in ATP synthesis and respiratory metabolism (Vicente et al., 2017).

### Protein-Coding Gene Annotation and General Characteristics

**FIGURE 3:**
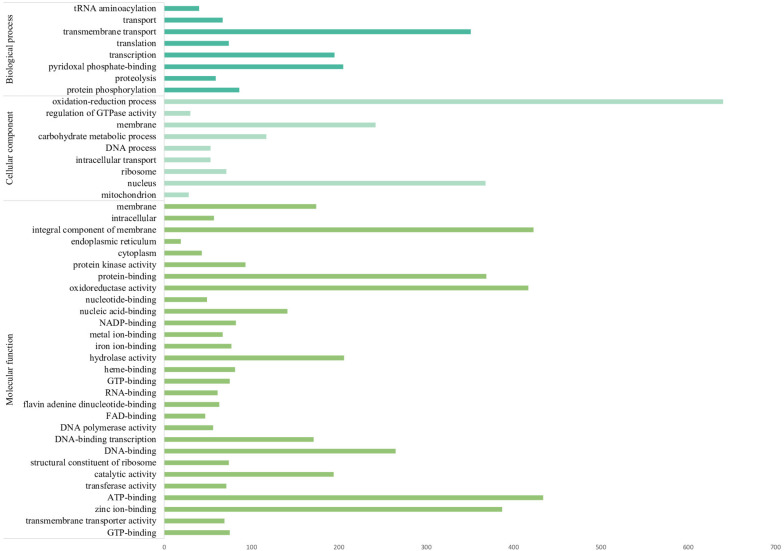
The most numerous gene families of *Fonsecaea pugnacius* based on Gene Ontology annotation separating proteins annotated into three large groups: biological process, cellular components, and molecular functions.

A total of 12,217 protein-coding genes were identified in *F. pugnacius*, of which 11,124 were annotated as hypothetical proteins and 1,093 proteins had inferred functions (Supplementary Table 1). In the GO annotation, the proteins were separated into three large groups: biological process, cellular components and molecular functions (Figure 3 and Supplementary Table 1). Among proteins to which functions were attributed, various proteins were shared among the *Fonsecaea* siblings, such as proteins related to virulence in transporter families, proteins from the glyoxylate cycle, genes encoding proteins related to oxidative stress and involved in the detoxification of reactive oxygen species (ROS), cytochrome P450 monooxygenases (CYPs/P450s), heat shock proteins, proteins of melanin pathways, enzymes able to degrade aromatic carbon compounds, and others.

Carrier families of zinc, iron, manganese, and sugar have also been identified, i.e., MFS transporters and ABC transporters. In addition to basal metabolism, these carriers play a role in survival strategies. MFS is the largest family of transporters, ubiquitous to all living organisms and involved in the active excretion of antifungal drugs (Vela-Corcía et al., 2019). Reportedly, they also enhance antifungal resistance in *Candida albicans*, *Aspergillus fumigatus*, and *Cryptococcus neoformans* (Costa et al., 2014).

Sequences of proteins involved in the glyoxylate cycle were annotated: isocitrate lyase and malate synthase (Supplementary Table 1). The glyoxylate pathway is a metabolic strategy for the synthesis of carbohydrates from carbon compounds, such as acetate and other degradation products from ethanol, fatty acids, and poly-*b*-hydroxybutyrate (White et al., 2017). The glyoxylate cycle consists of a modification of the citric acid cycle (TCA), as it shares the same initial reactions of citrate and isocitrate generation but continues with the formation of succinate and glyoxylate (Dunn et al., 2009). This pathway has been associated with fungal virulence, since it allows energy production in environments where complex carbon compounds are poorly found (Lorenz and Fink, 2001) and has been described in infectious fungi such as *Rhinocladiella mackenziei* (Moreno et al., 2018), *Fonsecaea* siblings related to chromoblastomycosis (Vicente et al., 2017), *Beauveria bassiana* (Yang et al., 2016), *Talaromyces marneffei* (Thirach et al., 2007), and *Candida albicans* (Lorenz and Fink, 2001).

In *Paracoccidioides brasiliensis*, agent of another implantation mycosis, paracoccidioidomycosis, an increase of transcriptional levels of isocitrate lyase and malate synthase genes was reported in an infection model (Derengowski et al., 2008). This suggests a possible mechanism of adaptation of the fungus in response to the internal environment of the phagosome, which is poor in complex sources of carbon. The same function could be assigned to *Fonsecaea* siblings to explain fungal persistence inside macrophages, which, according to Queiroz-Telles et al. (2017), seems to be fungistatic (rather than fungicidal) against agents of chromoblastomycosis. In addition, it may be related to the ability of these fungi to survive on low carbon sources during brain infection.

Characterization of the partial genome of *F. pugnacius* revealed genes encoding proteins related to oxidative stress and involved in the detoxification of ROS, such as alternative oxidase, manganese superoxide dismutase and cytoplasmic thioredoxins. Earlier reports observed that the alternative oxidase enzyme is present in the internal mitochondrial membrane of plants and some fungi and protozoa, in an alternative route of oxidation of the electron transport chain in cellular respiration (Duvenage et al., 2018). In the fungus *Neurospora crassa*, levels of the nuclear gene transcripts *AOX1*, which encode the alternative oxidase, increase when the cytochrome C oxidase pathway is inhibited. These results indicate activation of an alternative pathway, which the organism applies to correct conditions of oxidative stress and to decrease the production of ROS in respiration when competing with electrons of the classical oxidation pathway. Some studies have revealed that *AOX1* gene expression can be stimulated under stress conditions, such as low temperature and ROS low level, *AOX1* functioning as an antioxidant (Missall et al., 2004). Some pathogens resist to oxidative stress in the hostile environment of the phagosomes by the production of antioxidant enzymes that detoxify ROS, such as alternative oxidase, catalase and superoxide dismutase (Duvenage et al., 2018). ROS is an important cellular detrimental agent associated with the activation of immune response in human cells infected with fungi causing dermatomycoses or invasive mycoses (Castro et al., 2017).

According Vicente et al. (2017), many of the CYP/P450 enzymes revealed in herpotrichiellaceous fungi are abundantly present in *Fonsecaea* siblings. Likewise, they were observed in *F. pugnacius*. Cytochrome P450 monooxygenases are heme-thiolate proteins with roles in oxidative functions, e.g., degradation of xenobiotic compounds (Jawallapersand et al., 2014). Teixeira et al. (2017) noted that some black fungi are among the species of Ascomycota with the highest numbers of CYPs, with family expansion and diversification through gene duplication which might explain opportunism. The authors noted that these enzymes are involved in the metabolism of phenolic compounds and aromatic hydrocarbons, and suggested that similar compounds present in the human brain might explain their neurotropic predilection. Studies showed that fungi belonging to the genera *Fusarium*, *Penicillium*, *Aspergillus*, and the family Herpotrichellaceae are capable of degrading aromatic compounds (Conceição et al., 2005; Satow, 2005; Teixeira et al., 2017). Several species of black fungi have been isolated from hydrocarbon-contaminated environments (de Hoog et al., 2004). Fungi that are capable of assimilating monoaromatic hydrocarbons are enriched in the domestic environment (Sterflinger and Prillinger, 2001; Woertz et al., 2001; Prenafeta-Boldú et al., 2002). Prenafeta-Boldú et al. (2006) suggested physiological links between hydrocarbon assimilation by black fungi and certain patterns of brain infection. The brain contains small molecules that resemble alkylbenzene, phenylalanine metabolic products and lignin biodegradation intermediates, having structural similarity to neurotransmitters such as dopamine, which is catabolized in the brain (Fernstrom and Fernstrom, 2007). Tyrosine, used for the biosynthesis of the neurotransmitters dopamine, noradrenaline and adrenaline, has phenylalanine as precursor and it is involved in the formation of melanin and neuromelanin, dark pigments synthesized from L-dopamine for brain protection (Teixeira et al., 2017; Moreno et al., 2018).

Heat shock proteins are considered virulence factors because of their roles in thermotolerance and as molecular chaperones and are found in all prokaryotes and eukaryotes. They are classified based on approximate molecular weight (Tiwari et al., 2015). In *F. pugnacius*, the following families were identified: Hsp7, Hsp60, Hsp70, Hsp80, and Hsp90. Factors triggering the synthesis of heat shock proteins are oxidative or nutritional stress, UV radiation and exposure to chemical substances, indicating a protective role and aiding in cellular adaptation (Pockley, 2001).

Melanins confer resistance to heat, cold, enzymatic action and organic solvents, function as antioxidants and increase antifungal resistance (Nosanchuk and Casadevall, 2003). Main production route in *Fonsecaea* is the DHN pathway from acetate (acetyl-CoA) derived from glucose metabolism (Cunha et al., 2005; Casadevall and Eisenman, 2012). Melanin is a recognized virulence factor in several black and white pathogenic and opportunistic fungi, such as *Candida albicans, Cryptococcus neoformans*, *Aspergillus fumigatus*, *Exophiala dermatitidis*, *Paracoccidioides brasiliensis*, *Histoplasma capsulatum*, and *Sporothrix schenckii* (Jacobson, 2000; Langfelder et al., 2003; Morris-Jones et al., 2003; Nosanchuk and Casadevall, 2003). Homologous proteins were identified in the herpotrichiellaceous black fungi *Exophiala dermatitidis* (Youngchim et al., 2004). In *F. pugnacius*, melanin-associated proteins related to DHN and DOPA pathways were observed (Supplementary Table 1), as reported previously in *F. monophora* (Liu et al., 2019), such as tyrosinase, homogentisate dioxygenase and scytalone. Histopathological studies of organs such as brain, lung, liver and spleen in animal models did not clarify how the melanin production pathways are blocked in albino mutants of *E. dermatitidis* (Sudhadham et al., 2008), but the ability to block the oxidative burst increases significantly the pathogenic potential of *E. dermatitidis*, since the host is unable to eliminate this fungus (Kumar et al., 2019).

### Virulence in Animal Models

The chromoblastomycosis agent *F. pugnacius* was described causing a secondary disseminated infection in an apparently immunocompetent patient. Among all agents of chromoblastomycosis, this is a unique type of infection, starting with a chronic skin disease and finally evolving to cerebritis. de Azevedo et al. (2015) reported that *F. pugnacius* was able to produce muriform cells in skin, but hyphae were present in the brain. Dissemination from skin to brain apparently led to conversion to another type of invasive morphology which has not been observed in agents of chromoblastomycosis. In *F. monophora*, which has also been reported from brain infection, the infection route was probably by inhalation, as no skin involvement was observed in any of the patients. Brain infection with a subcutaneous origin has thus far only been observed in *F. pugnacius* (de Azevedo et al., 2015).

**FIGURE 4:**
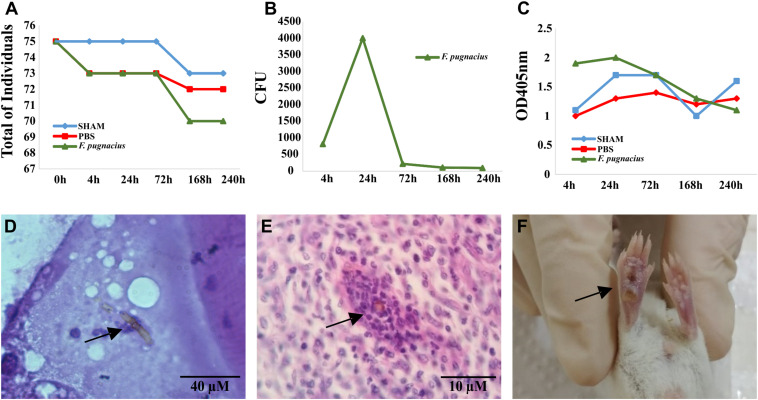
Virulence test of *Fonsecaea pugnacius* using animal models: *Tenebrio molitor* and Balb/c (*Mus musculus*). **(A)** Mortality of the *Tenebrio molitor* larvaes at intervals of 4, 24, 72, 168, and 240 h post-infection. **(B)** Fungal burden of *Tenebrio molitor* larvae tissues at intervals of 4, 24, 72, 168, and 240 h post-infection. **(C)** Melanization of *Tenebrio molitor* hemolymph demonstrated by measuring the OD405 nm at 4, 24, 72, 168, and 240 h post-infection. **(D)** Presence of melanized hyphae in *Tenebrio molitor* larvae tissue 72 h post-infection. **(E)** Histopathology of footpad Balb/c tissue with presence of muriform cell after 21 days of intradermal infection. **(F)** Necrotic lesion in footpad of Balb/c mice after 7 days of intradermal inoculation.

*Tenebrio molitor* larvae were infected with inoculum concentrations of 5 × 10^6^ cells/mL and observed for 10 days. The larvae infected with *F. pugnacius* exhibited higher mortality rates than control groups, PBS and SHAM (Figure 4A). *Fonsecaea pugnacius* presented a lower mortality rate than *F. monophora*, *F. erecta*, and *F. pedrosoi*, as reported by Fornari et al. (2018). This indicates that *F. pugnacius* infection presents a slower development compared to *Fonsecaea* siblings involved in chromoblastomycosis, as well as to environmental saprobes. The fungal burden inside the larvae was assessed and presented significant numbers of CFUs, despite the low mortality rates caused by *F. pugnacius*: CFU values were initially countless and decreased along 72, 164, and 240 h post-infection (Figure 4B).

Melanization of the larvae is an intracellular defense response and an effective barrier to infection. After 24 h, the larvae infected with *F. pugnacius* presented a dark pigmentation, caused by melanization in the hemolymph, which was observed during the entire 10-day period of analysis by visual observation and spectrophotometry (Figure 4C). Similar results were obtained by Fornari et al. (2018) in *Fonsecaea* siblings, showing maximum melanization with 24 h post infection. The capacity of the fungus to survive inside the larvae was confirmed by histopathology, revealing melanized hyphae in tissue that had developed within 4–72 h (Figure 4D).

Virulence tests using murine models Balb/c and C57/BL6 were conducted using two infection pathways: intradermal (per hind footpad) and intraperitoneal. In view of determination of fungal burden, *F. pugnacius* was recovered from kidney, lung and liver after 7 days of incubation, indicating a certain preference of the fungus for these organs. After 14 days of intraperitoneal inoculation, *F. pugnacius* was recovered from the brain. At 21 days after infection, a muriform cell was observed in histopathology of the footpad (Figure 4E). The animal host infected intraperitoneally presented 1 × 10^2^ and 1 × 10^4^ CFU/mL in blood and organs (lung, kidney and spleen) after 7 and 14 days, respectively. The animals infected intradermally presented 2 × 10^6^ CFU/g in the plantar cushion. The clinical aspects of these animals were evaluated, but no lesions, tissue necrosis or morphological alterations of internal organs were observed, except for plantar cushion swellings with (sub)cutaneous lesions (Figure 4F). Vicente et al. (2017) obtained similar results with Balb/c mice infected with *F. pedrosoi* by intradermal inoculation.

Immunological assays revealed insignificant levels of IL-2, INF-γ, TNF-α, IL-6 and IL-10, compared to what was described for other chromoblastomycosis agents including *F. pedrosoi* (Dong et al., 2018) and *F. monophora* (Jiang et al., 2018). The low immune response could be associated with a low virulent ability of *F. pugnacius*. Although the fungus carries several genes with roles in pathogenicity and ability to survive in murine tissue, the immune system may not recognize their protein products, judging from absence of a cytokine response.

### Potential Virulence Related to Enzymatous Genes

**FIGURE 5:**
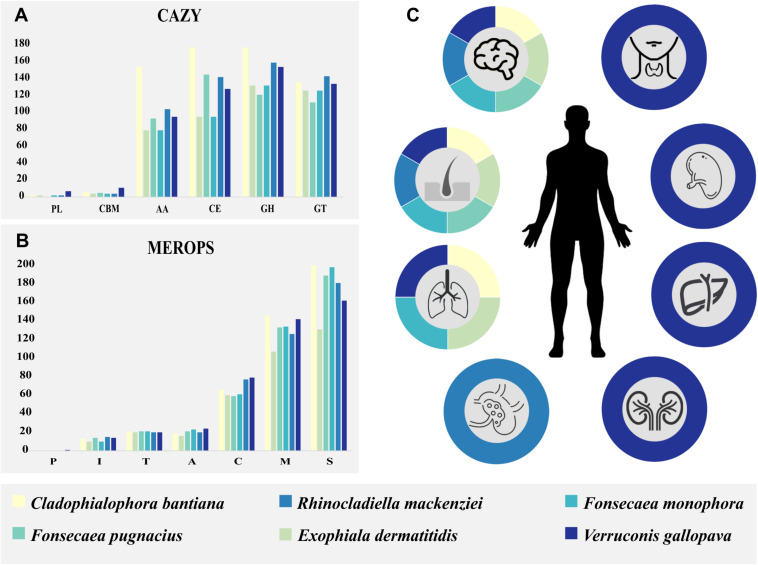
Species enzymatous gene profiles and epidemiology of human host pathogenicity are summarized in colored boxes. *Cladophialophora bantiana*: beige; *Exophiala dermatitidis*: light green; *Fonsecaea pugnacius*: medium green; *Fonsecaea monophora*: light blue; *Rhinocladiella mackenziei*: medium blue; *Verruconis gallopava*: dark blue. **(A)** Carbohydrate-active enzyme (CAZY) annotation separated by classes. **(B)** MEROPS annotation separated by classes. **(C)** The species of black fungi related to subcutaneous and cerebral human infection and others affected organs: lungs, lymphatic system, kidneys, spleen, liver, and thyroid gland.

The observed brain infection might have been enhanced by the fungus ability to metabolize monoaromatic substrates as carbon source. Among these compounds, vanillic acid, phenyl acid, L-tyrosine, L-dopa, L-phenylanine, dopamine, sphingosine, and others are present in mammalian brain (Prenafeta-Boldú et al., 2006). Subsequently, we verified whether an enzymatic apparatus competent to degrade these compounds is present in the *F. pugnacius* genome, i.e., with carboxylases, reductases, aldolases, and kinases. The enzymatous gene profile was established with CAZymes and MEROPS databases. Carbohydrate-active enzyme analysis resulted in 476 genes encoding putative CAZymes, comprising 93 auxiliary activities (AA), 5 carbohydrate binding modules (CBM), 145 carbohydrate esterases (CE), 121 glycoside hydrolases (GH), 112 glycosyl transferases (GT), and zero polysaccharide lyases (PL). For MEROPS, 21 aspartic peptidases (A) were annotated, 59 cysteine peptidases (C), 14 trypsin peptidases (I), 133 metallo-peptidases (M), zero glutamic peptidases (G), zero asparagines peptidases (N), zero mixed peptidases (P), 189 serine peptidases (S) and 21 threonine peptidases (T) (Figure 5).

We performed a comparative analysis of *F. pugnacius* with closely related neurotropic species: *Cladophialophora bantiana*, *Exophiala dermatitidis*, *Fonsecaea monophora*, *Rhinocladiella mackenziei*, and *Verruconis gallopava* (Figure 5 and Supplementary Table 2), which have been reported as agents of cutaneous and deep infection in skin, lungs, lymphatic system, kidneys, spleen, liver and thyroid glands (Cardeau-Desangles et al., 2013; de Azevedo et al., 2015; Geltner et al., 2015; Jennings et al., 2017; Mukhopadhyay et al., 2017; Stokes et al., 2017; Grewal et al., 2018; Sideris and Ge, 2018; Klasinc et al., 2019; Miossec et al., 2019; Wang et al., 2019; Alabdely and Alzahrani, 2020). The CAZy database is an exceptional reporter of fungal lifestyles degrading complex polysaccharides (Cantarel et al., 2009; Vicente et al., 2017). The GH class contains the majority of catalytic enzymes to degrade lignocelluloses (Cantarel et al., 2009) and seems to be expanded in species associated with disseminated infection (Figure 5). Through this analysis (Supplementary Table 3), we observed a high number of GH enzymes in all genomes analyzed, except for GH32 which was present in low numbers (*C. bantiana*: 3; *F. pugnacius*: 2; *E. dermatitidis*, *F. monophora*, *V. gallopava*: 1, *R. mackenziei*: 0). The CAZyme family GH32 are enzymes associated with energy storage and seem to provide energy for survival in extreme environments (Oren, 2011). Additionally, the GH18 family, observed in the species under study, are chitinases. β-1,3-Glucanases are able to degrade chitin that is normally present in animal exoskeletons. Likewise, 29 GH families involved in the degradation of plant biomass (Li et al., 2011) were observed in the strains evaluated, explaining the dual ability of these fungi to invade plant and animal tissues.

The high number glycosyltransferases (GTs) can also be linked to neurotropism, since these enzymes are responsible for the biosynthesis of glycoside (Cantarel et al., 2009). Some fungi are able to convert phenolic compounds into their corresponding glycosides (Tronina et al., 2013) and this ability may be derived from xenobiotic metabolism (Yang et al., 2014). The vertebrate brain contains a wide variety of gangliosides that are localized in specific cell types, such as on the surface of plasmatic membranes (Stanley, 2016). Gangliosides contain a fatty acid and a sphingosine base and are involved in neural functions such as memory formation, synaptic transmission, regeneration (Zitman et al., 2010). In cases of cerebral infection, the first symptoms described are locomotor difficulties and severe headache (Koo et al., 2010; de Azevedo et al., 2015). The GT41 family was the most numerous in our annotation, it is related to the metabolism of serine-threonine as indicated and involved in the process of subcutaneous infection (Vicente et al., 2017). Also, the GT2 family was abundant; proteins that act as chitin synthases (Breton et al., 2006) are numerous in many fungi (Lee et al., 2018; Stone et al., 2018).

The AA CAZyme family is composed of lignolytic enzymes and are commonly found in plant pathogens. Similar to the GH and CMB families, they have a role in breaking down plant cell-wall polysaccharides (Lowe et al., 2015). The CBM family is composed of lectins and sugar transporters, while GH are glycosyltransferases such as lignocelluloses or chitinases (Yang et al., 2014). However, the GH class was more numerous in the species related to disseminated and cerebral infection (Figure 5). The lectins from yeasts and fungi have been associated with early stages of human infection (Varrot et al., 2013), whereas in bacteria they seem to be involved in recognition of host glycans (Imberty and Varrot, 2008).

Polysaccharide lyases are enzymes able to cleave polysaccharide chains. This group presents many fold types (or classes), indicating that PLs are polyphyletic (Lombard et al., 2010). Many fungi that do not have enzymes from the PL CE and GH families are saprobes, as these classes have enzymes related to cell wall degradation in plants (Zhao et al., 2013). This enzymatous gene profile suggested a dual ecological ability of these agents, in line with their extremotolerance and adaptability to variable environmental niches, which is a prerequisite for their opportunistic profiles.

Peptidases play key roles in penetration of microorganisms into host tissue and are involved in pathogen-host interactions (Ohm et al., 2012). Herpotrichiellaceous agents produce a variety of extracellular peptidases for the degradation of environmental substrates, indicating a poorly specialized nutritional strategy (Sriranganadane et al., 2010; Vicente et al., 2017). The MEROPS S (serine) and M (metallo) peptidase families were the highest in number in the analyzed species causing brain infection. These two groups have been reported to be significantly enriched in transcriptome analyses of *E. dermatitidis* during infection (Poyntner et al., 2018). Among the Serine families, classes S33 and S9 were more numerous (Supplementary Table 3), both involved in prolyl metabolism. Class S33 is a prolyl aminopeptidase family, which is not essential for growth but may confer a selective advantage allowing the organism to use proline-rich substrates (Iqbal et al., 2018). Teixeira et al. (2017) observed an expansion of the protein-degrading peptidase enzyme family M38 (isoaspartyl dipeptidases) in the bantiana-clade, the most numerous of metallo-peptidases family in the analyzed strains. This family is unusual in that the majority of characterized proteins are not peptidases but are associated with β-aspartic dipeptidase acting in the release of iso-aspartate residues from peptides (Palmeira et al., 2018).

Differences between closely related taxa are expected in ecology-related genes. The enzymatic repertoire of these fungi shows their ability to degrade a wide variety of substrates (Figure 5). This may suggest generalist and opportunistic ecology comparable to *Aspergillus* spp. permitting transfer from the environment to the animal host (Vicente et al., 2017), rather than pathogenicity where focused adaptation (Moran et al., 2011). In addition, we analyzed genes codifying proteins related to degradation aromatic carbons pathway (Teixeira et al., 2017; Moreno et al., 2018) and the strains studied present a range of genes encoding homologous proteins (Table 2). The virulence of these strains is partially explained by general factors like the presence of melanin in the cell wall, thermotolerance and the ability to assimilate of monoaromatic hydrocarbons (Moreno et al., 2018). Furthermore, CYPs are involved in the degradation of aromatic hydrocarbons and xenobiotic metabolism, developing functions in the fungal pathogenicity and in the detoxification of exogenous compounds (Moreno et al., 2018).

**TABLE 2:** Proteins related to degradation aromatic carbons pathway in *Fonsecaea pugnacius* and homologs in black fungi related to subcutaneous and brain infection.

	**A**	**B**	**C**	**D**	**E**	**F**
*Fonsecaea pugnacius*	gi| 628298155		gi| 915117215	gi| 915113179 gi| 915117218	gi| 915082939	gi| 915113183 gi| 915117846 gi| 915047106 gi| 915117212 gi| 915114200
*Cladophialophora bantiana*		XP_016622135.1	XP_016621839.1 XP_016624089.1 XP_016620669.1	XP_0166207751 XP_0166218381	XP_016619449.1	XP_016625963.1 XP_016624090.1 XP_016621840.1
*Exophiala dermatitidis*		HMPREF1120_09097	HMPREF1120_02976 HMPREF1120_03465 HMPREF1120_03826	HMPREF1120_ 03827	HMPREF1120_03438	HMPREF1120_ 03825
*Fonsecaea monophora*	AYO21_00136 AYO21_09571	AYO21_05363 AYO21_04106	AYO21_12165	AYO21_04852 AYO21_01974	AYO21_00578	AYO21_07675 AYO21_04851
*Verruconis gallopava*	PV09_08030		PV09_06763	PV09_04037 PV09_05381 PV09_02442		PV09_02443 PV09_02062
*Rhinocladiella mackenziei*		Z518_08486	Z518_05387	Z518_05995 Z518_05388 Z518_06895 Z518_09726	Z518_00072	Z518_02618 Z518_04953 Z518_04319 Z518_05993 Z518_05386 Z518_09725

*(A) Benzyl alcohol dehydrogenase, **(B)** β-carboxy-*cis*, *cis*-muconate lactonizing enzyme, **(C)** phenylacetate 2-hydroxylase, **(D)** homogentisate 1,2-dioxygenase, **(E)** maleylacetoacetate isomerase, **(F)** fumarylacetoacetase.*

The family Herpotrichiellaceae contains numerous black fungi that present tolerance to various types of stress, showing great adaptability to extreme environmental conditions, presumably resulting from genomic information. Genomic studies of *F. pugnacius* showed the wide variety of genes involved in extreme tolerance and enzymes associated to occurrence of virulence factors. The survival capacity of fungi in animal models was confirmed by histopathological analysis and the presence of melanin in the host tissue. We have shown that *F. pugnacius* can colonize the brain and cause subcutaneous lesions with the formation of muriform cells in the murine model. An ecological capacity can be concluded from the presence of metabolic pathways for extremetolerance combined with the ability to infect human hosts. However, complementary molecular studies must be carried out in order to strengthen the connections between ecology and clinical profiles.

Gostinčar et al. (2018) found a link between (poly-)extremotolerance and opportunism, the ability to metabolize monoaromatic hydrocarbons improving human or animal disease. The order Chaetothyriales comprises opportunists, in which tolerance to various types of stress is associated with adaptability, presumably resulting in a large potential for habitat changes. The infection is as a side effect of the fungus adaptation to the human host, demonstrating that is not a favorable habitat, nor relevant to their evolutionary process. This defines opportunism against pathogenicity, where the infection is advantageous for the fitness of the species. Most organisms considered opportunistic are unable to transmit from host to host, so specific adaptations will be lost with the cure of the infection, explaining the lack of complex virulence characteristics. Therefore, opportunistic infections can be considered an evolutionary dead end, which is unlikely to lead to true pathogenicity (Gostinčar et al., 2018).

## Data Availability Statement

The datasets generated for this study can be found in the GenBank/accession number: WJFF00000000/waiting to publish for to be public date.

## Ethics Statement

All animal experiments in this study were approved by the Federal University of Paraná Ethics Committee (approval certificate 1002) and performed according to the Committee’s recommendations.

## Author Contributions

AB, VV, RG, and SH contributed to the conception and design of the study. FC, AL, LM, and NS organized the database. AB, GS, BS, FM, BL, RC, VB, EB, VP, and NH performed the analysis. AB wrote the first draft of the manuscript. GS wrote sections of the manuscript. All the authors contributed to the manuscript revision, read and approved the submitted version.

## Conflict of Interest

The authors declare that the research was conducted in the absence of any commercial or financial relationships that could be construed as a potential conflict of interest. The reviewer AM declared a past co-authorship with one of the authors SH to the handling editor.

## Footnotes

1 http://www.bioinformatics.babraham.ac.uk
